# Vitamin D, folate, and potential early lifecycle environmental origin of significant adult phenotypes

**DOI:** 10.1093/emph/eou013

**Published:** 2014-04-02

**Authors:** Mark Lucock, Zoë Yates, Charlotte Martin, Jeong-Hwa Choi, Lyndell Boyd, Sa Tang, Nenad Naumovski, John Furst, Paul Roach, Nina Jablonski, George Chaplin, Martin Veysey

**Affiliations:** ^1^School of Environmental and Life Sciences, ^2^Biomedical Sciences and Pharmacy, ^3^Maths and Physical Sciences, University of Newcastle, PO Box 127, Brush Road, Ourimbah, NSW 2258, Australia, ^4^The Pennsylvania State University, Anthropology Department, 409 Carpenter Building, University Park, PA 16802, USA, and ^5^Teaching and Research Unit, Central Coast Local Health District, PO Box 361, Gosford, NSW 2250, Australia

**Keywords:** vitamin D, folate, VDR, solar cycle, UV, UV-R exposure, photoperiod, insulin, hypertension, thymidylate synthase, serine hydroxymethyltransferase, melanization, pigmentation

## Abstract

**Background and objectives:** Vitamin D and folate are highly UV sensitive, and critical for maintaining health throughout the lifecycle. This study examines whether solar irradiance during the first trimester of pregnancy influences vitamin D receptor (VDR) and nuclear folate gene variant occurrence, and whether affected genes influence late-life biochemical/clinical phenotypes.

**Methodology:** 228 subjects were examined for periconceptional exposure to solar irradiance, variation in vitamin D/folate genes (polymerase chain reaction (PCR)), dietary intake (food frequency questionnaire (FFQ)) and important adult biochemical/clinical phenotypes.

**Results:** Periconceptional solar irradiance was associated with VDR-BsmI (*P* = 0.0008^wk7^), TaqI (*P* = 0.0014^wk7^) and EcoRV (*P* = 0.0030^wk6^) variant occurrence between post-conceptional weeks 6–8, a period when ossification begins. Similar effects were detected for other VDR gene polymorphisms. Periconceptional solar irradiance was also associated with 19 bp del-DHFR (*P* = 0.0025^wk6^), and to a lesser extent C1420T-SHMT (*P* = 0.0249^wk6^), a folate-critical time during embryogenesis. These same genes were associated with several late-life phenotypes: VDR-BsmI, TaqI and ApaI determined the relationship between dietary vitamin D and both insulin (*P* < 0.0001/BB, 0.0007/tt and 0.0173/AA, respectively) and systolic blood pressure (*P* = 0.0290/Bb, 0.0299/Tt and 0.0412/AA, respectively), making them important early and late in the lifecycle. While these and other phenotype associations were found for the VDR variants, folate polymorphism associations in later-life were limited to C1420T-SHMT (*P* = 0.0037 and 0.0297 for fasting blood glucose and HbA1c levels, respectively). We additionally report nutrient–gene relationships with body mass index, thiol/folate metabolome, cognition, depression and hypertension. Furthermore, photoperiod at conception influenced occurrence of VDR-Tru9I and 2R3R-TS genotypes (*P* = 0.0120 and 0.0360, respectively).

**Conclusions and implications:** Findings identify environmental and nutritional agents that may interact to modify gene–phenotype relationships across the lifecycle, offering new insight into human ecology. This includes factors related to both disease aetiology and the evolution of skin pigmentation.

## Abstract (precis)

Solar radiation early in pregnancy interacts with light sensitive vitamins to influence an embryo's genetic profile. This influences both adult disease risk and may play a role in the evolution of skin colour.

## INTRODUCTION

Vitamin D and folate are highly UV sensitive, and critical for maintaining health throughout the lifecycle. We examine whether solar irradiance during the first trimester of pregnancy influences the vitamin D receptor (VDR) gene and nuclear folate gene variant occurrence, and whether affected genes influence late-life biochemical/clinical phenotypes. Findings are examined in the context of both the early origins of adult disease and the evolution of skin pigmentation.

The UV-dependent vitamin D endocrine system plays a major regulatory role in human biology. It controls bone and calcium homeostasis, regulates cell proliferation and differentiation and modulates the immune response [1]. Perturbations in this endocrine system are thus linked to a wide range of common diseases that include cancers, cardiovascular disease (CVD) as well as diabetes [2]. Indeed, low vitamin D alters insulin synthesis and secretion [3] and is associated with obesity [4].

Vitamin D synthesis involves the UV-B (280–320 nm) conversion of 7-dehydrocholesterol into previtamin D_3_ in the epidermis of the skin. Previtamin D_3_ can then photoconvert to lumisterol or tachysterol (inactive metabolites), or undergo temperature-dependent isomerization to vitamin D_3_ (cholecalciferol/calciol) which is then metabolized to calcidiol [25(OH)D_3_], and subsequently, on to calcitriol [1,25(OH_2_)D_3_] [5].

Expression and nuclear activation of the VDR gene are required to mediate the signalling effect of vitamin D obtained from the diet or via its synthesis in the skin by UV-B radiation. The VDR requires its unliganded heterodimer retinoid X-receptor partner for transactivation [6]. Many polymorphisms exist in the VDR gene, which regulates 3% of the human genome. Examples of variants that are located between exons 8 and 9 include Tru9I, TaqI, BsmI, EcoRV and ApaI [7–10], while FokI occurs in exon 2 [11, 12]. These variants are associated with altered risk for several important clinical phenotypes, most often degenerative disorders; however, it also plays a role early in the lifecycle with VDR being present in reproductive tissues of both genders [13]. Crucially, it may play a role in fertility [13–16]. Levels of 25(OH)D_3_ show a marked seasonal variation with a post-summer high and post-winter low well established at latitudes beyond 40° N and S of the equator. Limited evidence indicates vitamin D may influence *in vitro* fertilization [13, 14] and that it is aetiologically significant in polycystic ovary syndrome [17–20] where it may play a role in insulin resistance and the metabolic syndrome. Furthermore, vitamin D metabolism is critical for several developmental processes during early pregnancy [20], including foetal bone health [21], with foetal ossification beginning around post-conception week 7.

Within the cell, folate coenzymes are required for biosynthesis of DNA-thymidylate (dTMP) and *de novo* generation of methionine for both genomic (CpG) and non-genomic methylations [22]. They are also required for purine synthesis and serine–glycine interconversions as well as histidine catabolism [23]. DNA fragility occurs in folate depletion resulting from uracil being misincorporated into the primary base sequence in place of thymine. Folate originated methionine biosynthesis contributes methyl groups to the methylome and hence governs gene expression. Half the methionine requirement is met via the folate *de novo* route [24, 25]. Perturbations in folate nutrition and/or genetics are associated with neural tube defects (NTDs) and a vast swathe of other developmental and degenerative disorders, including cancers and vascular disease [26].

Given the UV dependency of vitamin D signalling as an important determinant of phenotype throughout the lifecycle, and following the recent discovery that periconceptional UV-R predicts folate-sensitive, epigenomic-related neonatal genotypes (MTHFR and MSR) during the first trimester of pregnancy [27, 28], we examined the solar cycle-related irradiance for each post-conceptional week through the first trimester to see if this surrogate for UV-R might additionally alter VDR polymorphism frequency in the same way [28]. We also examined whether solar cycle fluxions during embryogenesis and early foetal development might influence gene variant frequency within the specific gene cluster encoding serine hydroxymethyltransferase (SHMT1), thymidylate synthase (TS) and dihydrofolate reductase (DHFR). These genes operate in a tight synergy and are fundamental to the fidelity of DNA (dTMP) biosynthesis, making them critically important during early embryo development and throughout the first trimester. These folate enzymes are post-translationally modified to allow nuclear translocation during S and G2/M cell cycle phases [29, 30].

Given recent interest in vitamin D and health, we were further prompted to see if any VDR gene variants that were affected by the solar cycle could subsequently modify health-related phenotypes in adult life—a true cradle to grave evaluation of vitamin D. Folate, by comparison, is far better characterized in terms of its known health correlates [26], although an examination of folate gene variants and later life phenotypes was still carried out.

Solar eruptions can lead to charged particles affecting satellites, communications and power grids. Considerable work has focused on the impact of these solar events on human infrastructure, but little work has looked at the effects on human biology. Total solar irradiance (TSI), which increases with sunspot activity [31], is a balance between sunspot-related magnetic forces shielding the solar plasma, and highly energetic faculae that surround sunspots.

Since UV-A has better penetration of both ozone and human skin than does UV-B/C [27, 31–35], it is likely to be a highly relevant component of the TSI in the context of vitamin D stability and photodegradation: Vitamin D_3_ synthesized in the skin by the action of UV-B can be degraded by UV-A after only 10 min of non-tropical sun, although the rate of loss is lower in winter [5, 36].

Similarly, folate exhibits an important relationship with UV light. A proposition that dermal exposure to UV-A radiation promotes folate photolysis and lowers folate status has been made [35, 37, 38] and is supported by a more recent Australian study [39]. Near UV-A light at 312 nm can degrade plasma/cellular 5-methyltetrahydrofolate (5CH_3_-H_4_folate), leading to formation of labile, oxidized 5-methyldihydrofolate (5CH_3_-H_2_folate), with eventual, irreversible loss of all vitamin activity via C9–N10 bond scission [37]. This has led to the suggestion that one of the factors driving the evolution of skin pigmentation was the need to protect labile folate vitamers, which are critical for reproductive success, from UV-A radiation (the folate–vitamin D-sunlight hypothesis), a paradigm first enunciated by Jablonski [35] and Jablonski and Chaplin [40]. The theory links UV-labile 5CH_3_-H_4_folate and the light-sensitive hormone vitamin D with ambient UV exposure as factors in the evolution of skin pigmentation and depigmentation, respectively, although numerous genes are now also known to be important [41–43]. In effect, it is proposed that natural selection maintains two opposing phenotypic clines of skin pigmentation. The first of these relates to vitamin D (cholecalciferol) photosynthesis, involving a graded shift from high to low pigmentation as one moves from equator to pole. The second cline relates to photolytic C9–N10 bond scission of 5CH_3_-H_4_folate, where skin pigmentation protects in a graded manner from low to high protection as one moves along the same longitudinal sweep, but this time from pole to equator. Between clinical extremes of UV exposure, facultative melanization has evolved to handle seasonal flux in UV exposure. Interestingly, the genetic maximal level of constitutive pigmentation occurs in the late teens/early 20 s when the period of peak fertility and hence maximal folate requirement occurs [44, 45].

It has been suggested that the photoperiod a woman is exposed to at conception can influence the 677T-MTHFR allele frequency of her offspring based on the hypothesis that longer days promote greater UV-A-related folate loss during the periconceptional period, and therefore create a greater need for an embryo to possess the T allele in order to maintain DNA fidelity via *de novo* dTMP synthesis [27]. This seems to support the ‘folate–vitamin D-sunlight hypothesis’ [35, 40], as does a similar finding in which the intensity of solar radiation reaching Earth at conception influences both C677T-MTHFR and A66G-MSR genotype occurrence [28]. While folate-dependent DNA synthesis and maintenance of the epigenome are both critical during embryogenesis [46], photo-sensitisers like vitamin B_2_ [47] may also influence embryo viability by enhancing UV-dependent folate degradation [27, 40]. Variant folate-related genes can be sensitive to systemic levels of this vitamin [48]; when such genes maintain cell differentiation and growth because they encode proteins needed for epigenetic methylations and/or the regeneration of folyl vitamers required for dTMP synthesis, any factor that diminishes systemic folate status could selectively terminate embryos carrying these specific variant genes. Indeed, the rate of pregnancy loss *per se* after conception is estimated to be as high as 70–80%. This new paradigm may help explain aspects of early pregnancy loss and the origin of clinically relevant phenotypes based on UV-R exposure, folate lability and key DNA/methylome-related genetic polymorphisms. It also raises interesting questions on the ‘folate–vitamin D-sunlight hypothesis’. The present study further explores this idea in the context of folate and looks at similar relationships involving vitamin D.

We have examined a key environmental influence (periconceptional solar cycle UV-R) on vitamin D and folate to see if early lifecycle events can leave a signature on later life adulthood disease, a process that might be modulated by VDR gene variants and three folate genes which are critical to the elaboration of DNA. Indeed, there is increasing evidence that human biology and clinical phenotypes are influenced by geophysical cycles related to early life events [49], with the solar cycle recently linked to occurrence of multiple sclerosis [50], a condition often associated with vitamin D status [51]. Eight important biochemical phenotypes and five clinical phenotypes are examined in the present study. All are considered to be important markers of healthy ageing. Finally, the paper considers whether there is any evidence from this study to support and build upon the ‘folate–vitamin D-sunlight hypothesis’ of skin pigmentation first put forward by Jablonski and Chaplin [40].

Drawing attention to environmental and nutritional agents that modify gene–phenotype relationships across the lifecycle may provide important new insight into human ecology.

## METHODOLOGY

### Subjects and sample collection

Two hundred twenty eight volunteers from New South Wales—Central Coast retirement villages (65–96 years, 91 males and 137 females) were assessed for the prevalence of eight VDR polymorphisms, vitamin D intake, indices of (i) vascular health, insulin, fasting glucose, glycosylated haemoglobin (HbA1c), body mass index (BMI), aminothiols (homocysteine, cysteine and glutathione), red cell folate, systolic and diastolic blood pressure (hypertensive phenotype) and (ii) mental wellbeing; cognitive ability and trend towards a depressive phenotype using the Mini Mental State Examination (MMSE) and the Hospital Anxiety and Depression Scale (HADS), respectively. Blood was collected and processed as appropriate, and place and date of birth recorded. Informed consent was obtained prior to volunteers participating in the study under University of Newcastle Human Research Ethics Committee approval—H-782-0304 and Northern Sydney Central Coast Health Human Research Ethics Committee approval—04/19.

### Photoperiod and solar cycle activity

Photoperiod at conception has been calculated in minutes via Photoperiod Calculator V 1.94 L (http://www.sci.fi/∼benefon/sol.html). Additionally, for every subject the solar activity/irradiance has been recorded for each of 90 days following conception (equating to the first trimester) representing around 20 500 observation days [28]. Solar activity has been assessed via the Royal Greenwich Observatory—USAF/NOAA Sunspot Database (http://solarscience.msfc.nasa.gov/greenwch.shtml). The changing number of sunspots (as measured by the occluded area of the sun visible from Earth in units equating to millionths of a hemisphere) associated with an approximate 11.1 year sunspot cycle predicts the amount of solar radiation hitting Earth [28, 52]. Presumptive time of conception in this model assumes pregnancy lasts exactly 9 months, although clearly the presumptive time of conception will represent a narrow periconceptional window that includes implantation and very early embryogenesis.

### DNA analysis

VDR gene variants were examined using the polymerase chain reaction (PCR) to amplify blood DNA followed by restriction enzyme digestion and gel electrophoresis: VDR-Tru9I, VDR-TaqI, VDR-BsmI, VDR-EcoRV, VDR-ApaI, VDR-cdx2, VDR-NIaIII and VDR-FokI assays are based on the following methods [53–58]. The folate gene polymorphisms 19 bp del-DHFR, 2R3R-TS, 1494del6-TS and C1420T-SHMT were analysed according to the following methods [59–62].

### Haematology

Fasting plasma insulin (mIU/l) and percentage HbA1c, along with red cell folate were measured using routine hospital assays. The aminothiols (homocysteine, cysteine and glutathione) were analysed using HPLC: following derivatization with the fluorogenic reagent SBDF, gradient HPLC with fluorescence detection was used to measure plasma thiol levels according to an established method [63].

### Body mass index

BMI was calculated from height and weight data (BMI = weight/height^2^; kg/m^2^) as a standardized measure of body size for comparison and for use as a measure of overall adiposity (obesity is defined as a BMI >30 kg/m^2^ and overweight as a BMI of 25–29.9 kg/m^2^).

### Blood pressure

Blood pressure measurements were ascertained on three separate occasions over 6 months; the average being taken as the subject’s indicative blood pressure. At each clinic, blood pressure was measured in a recumbent position after resting for at least 5 min (recumbent blood pressure). A standard mercury sphygmomanometer was used and first (systolic) and fifth (diastolic) Korotkoff sounds were recorded to the nearest 2 mm Hg. Two sets of readings were taken and averaged for each clinic. Hypertension was diagnosed at an average systolic reading of >140 mm Hg and/or diastolic reading of > 90 mm Hg, and these were the values used to define clinical phenotype in this study [64].

### Psychometric analyses

#### Hospital Anxiety and Depression Scale

The HADS test is a 14-item questionnaire that assesses emotions during the previous week. It consists of two scales: an anxiety subscale (HADS A) and a depression subscale (HADS D), each with seven questions. Scores for each question are graded 0–3, and the aggregate scores for the seven questions for each subscale are used to gauge level of depression and anxiety. Aggregate scores for each subscale are also divided into categories defining the participant’s depression and anxiety level as ‘normal’ (0–7) ‘mild’ (8–10), ‘moderate’ (11–14) and ‘severe’ (15–21) [65, 66].

#### Mini-Mental State Examination

The MMSE provides a brief 30-point questionnaire to assess cognitive impairment. It is commonly used to screen for dementia, and also estimates severity of cognitive decline by following the course of cognitive changes in an individual over time. It takes around 10 min to evaluate arithmetic, memory and orientation functions [67].

A score of ≥25 points (out of 30) implies normal cognition. A score below this indicates severe (≤9 points), moderate (10–20 points) or mild (21–24 points) cognitive impairment [68]. Low scores correlate closely with dementia [69–71].

### Food frequency questionnaire for intake of Vitamin D

Estimated daily intake of vitamin D was assessed by interviewer administered food frequency questionnaire (FFQ). The questionnaire covered 225 food items and all food groups. Subjects also provided a list of all supplements they were taking, and were asked about these during the FFQ interview.

The FFQs were analysed using Foodworks^TM^ 2.10.146 (Xyris Software, Brisbane, QLD, Australia). The package uses food databases covering the majority of foods consumed by Australians, including AusFoods (brands), Aus Nut (base foods) and the New Zealand—Vitamin and Mineral Supplements 1999 databases. A more comprehensive description of the FFQ methodology is provided in Lucock et al. [72].

### Statistics

Statistical analysis was performed using JMP (version 9.0; SAS Institute Inc., Cary, NC, USA). The associations between key variables and related parameters assessed on an *a priori* basis were examined using either standard least squares analysis, or, where nominal/ordinal data were examined, logistic regression analysis that fits the cumulative response probabilities to the logistic distribution function of a linear model using maximum likelihood; *P*-value (*P* < 0.05) for the Wald χ^2^ test/effect likelihood ratio test (use as indicated), provided a significance marker for screening effects. Descriptive statistics were calculated and presented as appropriate.

Analysis with stepwise regression was performed in a mixed direction with significant probability [0.250] that a parameter be considered as a forward step and entered into the model or considered as a backward step and removed from the model. Mallow’s Cp criterion was used for selecting the model where Cp first approaches *P* variables. While an initial alpha level of 0.05 was set, a Bonferroni correction for multiple comparisons was additionally performed to identify the appropriate *P* value for significance based on the number of comparisons made, hence establishing whether significance was maintained.

## RESULTS

**Figure 1.**
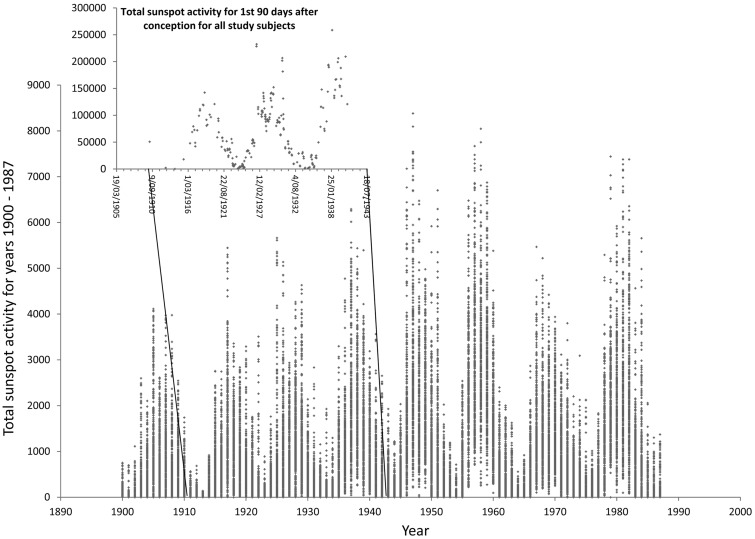
TSI for the first 90 days following the presumptive date of conception for each study subject. This has been plotted against the presumptive date of conception, which is superimposed against the TSI (total sunspot activity) for every day between 1900 and 1987.

The elderly population examined in this study was conceived over approximately three solar cycles. Total sunspot activity for the first 90 days following the presumptive date of conception (for each subject) has been plotted against presumptive date of conception in Fig. 1, which is superimposed against total sunspot activity (solar irradiance) for every day between 1900 and 1987.

### Vitamin D-related findings

Eight VDR gene variants were examined and are listed in Table 1, which shows where significant relationships existed between total sunspot activity and genotype for each week of the first trimester. The table gives the *P*-value (including *r*^2^ and the number of observations) for the effect likelihood ratio following stepwise regression and subsequent ordinal logistic modelling. Similarly, Table 2 shows where significant relationships existed between photoperiod at conception and VDR genotype occurrence.

**Table 1.** Significant relationships between total sunspot activity (TSI) and VDR gene variants for each week of the first trimester of pregnancy.^a^

Post-conception Week	VDR-TaqI	VDR-Tru9I	VDR-FokI	VDR-NIaIII	VDR-ApaI	VDR-BsmI	VDR-Cdx2	VDR-EcoRV
Week 1								
Week 2								
Week 3								
Week 4								
Week 5								
Week 6								*0.0030 (0.021, 204)
Week 7	*0.0014 (0.033, 214)				**0.0304** (0.010, 220)	*0.0008 (0.026, 207)		
Week 8	**0.0190** (0.033, 214)							
Week 9				**0.0289** (0.011, 225)				
Week 10		**0.0227** (0.031, 220)						
Week 11		**0.0139** (0.031, 220)						
Week 12								

^a^The table gives the *P*-value (including *r*^2^ and the number of observations) for the effect likelihood ratio test following stepwise regression analysis and subsequent ordinal logistic fit. An asterisk indicates where significance is maintained following a Bonferroni correction for multiple comparisons.

**Table 2.** Significant relationships between photoperiod at conception and occurrence of VDR genotype.^a^

	VDR-TaqI	VDR-Tru9I	VDR-FokI	VDR-NIaIII	VDR-ApaI	VDR-BsmI	VDR-Cdx2	VDR-EcoRV
Photoperiod at conception		**0.0120** (0.012, 212)	*0.0588 (0.008, 219)*					

^a^The table gives the *P*-value (including *r*^2^ and the number of observations) for the effect likelihood ratio test using ordinal logistic regression.

**Figure 2.**
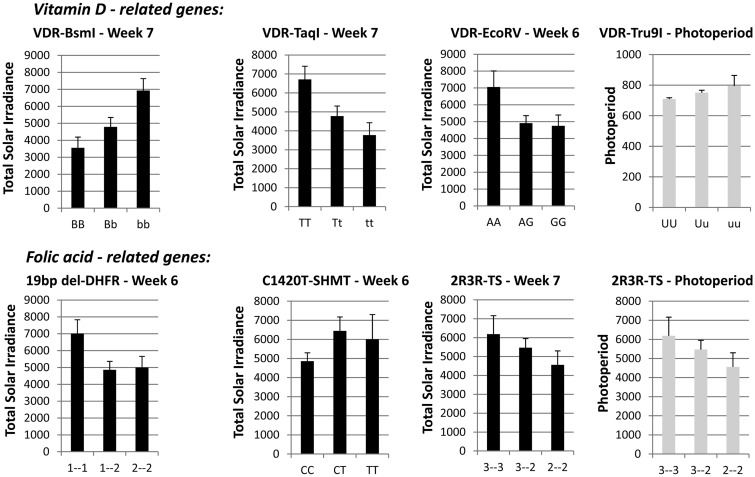
Top, effect of TSI and photoperiod on vitamin D-related genotype occurrence/mutant allele carriage for post-conceptional weeks 7 (VDR-BsmI and VDR-TaqI) and 6 (VDR-EcoRV). The figure also shows that VDR-Tru9I occurrence is related to photoperiod at conception. All graphs reflect a statistically significant association between TSI/photoperiod and genotype (effect likelihood ratio test *P* < 0.05). Bottom, effect of TSI and photoperiod on folate-related genotype occurrence/mutant allele carriage for post-conceptional weeks 6 (19 bp del-DHFR and C1420T-SHMT) and 7 (2R3R-TS). The figure also shows that 2R3R-TS occurrence is related to photoperiod at conception. Again, all graphs reflect a statistically significant association between TSI/photoperiod and genotype (effect likelihood ratio test *P* < 0.05). All graphs portray mean values and standard error of the means (SEM)

Table 1 shows that the most obvious effect of solar irradiance was on VDR-BsmI, VDR-TaqI and VDR-EcoRV. The key embryonic period of significant variation in VDR associated with UV-R occurred between post-conceptional weeks 6 and 11, the period when ossification of the skeleton begins, a physiologic process closely linked to vitamin D. While the quanta of UV given in Table 1 showed interesting effects on at least three VDR variants, the effect of the duration of light at conception given in Table 2 influenced variation in only one variant—VDR-Tru9I. Interestingly, VDR-Tru9I genotype was also associated with solar irradiance at post-conceptional weeks 10 and 11, although significance was not maintained following a Bonferroni correction for multiple testing. In terms of the VDR-BsmI, the exposure to solar irradiance increased with carriage of the mutant *b* allele in post-conceptional week 7. Figure 2 graphically illustrates this effect of mutant allele carriage for VDR-BsmI and TaqI at post-conceptional week 7 and VDR-EcoRV at post-conceptional week 6. These three relationships maintained significance following a Bonferroni correction for multiple testing with *P* = 0.0008, 0.0014 and 0.0030, respectively.

While these results reflect environment–gene interactions at the earliest phase of the lifecycle, Table 3 shows how the eight VDR gene variants and particularly those affected by the UV profile in the first trimester of pregnancy influenced important health phenotypes at the end of the lifecycle. Table 3 shows where any significant relationships existed between clinical and biochemical phenotypes and an individual’s VDR genotype. The table gives the *P*-value, regression coefficient, slope estimate and SE along with the number of observations for the appropriate regression model. Table 3 also shows whether dietary intake of vitamin D was predictive of these same important late-life phenotypes, but in a VDR genotype-dependent fashion. These statistical data are given in brackets with the specific genotype(s) that facilitated any significant association clearly indicated.

**Table 3.** Later life biochemical and clinical phenotypes associated with VDR variant genotype occurrence.^a^

Clinical/Biochemical Phenotype	VDR-TaqI	VDR-Tru9I	VDR-FokI	VDR-NIaIII	VDR-ApaI	VDR-BsmI	VDR-Cdx2	VDR-EcoRV
Insulin (mIU/l)	NS (**†tt 0.0007,** 0.354, 2.213, 0.575, *29***)**				NS (**†AA 0.0173,** 0.092, 0.565, 0.231, *61***)**	NS (**†BB <0.0001,** 0.380, 2.376, 0.528, *35***)**		
Fasting glucose (mmol/l)								
HbA1c (%)	*0.0668 (0.025, 213)*							
BMI		NS (**†Uu** *0.0509, 0.076, 0.151, 0.075, 51*)	**0.0076** (0.043, *227*)				NS (**†GG 0.0346,** 0.071, 0.130, 0.060, *63*)	
Homocysteine (µmol/l)								
Cysteine (µmol/l)		**0.0086** (0.043, *220*)						
Glutathione (µmol/l)					NS (**†aa 0.0280**, 0.099, 0.420, 0.185, *49*)			*0.0549 (0.028, 204)*
Red cell folate (nmol/l)								
*Cognition					NS (**δaa 0.0019** (0.061, *49*))			
*Depression					NS (**δAA, 0.0337, (**0.019, *61***))**		NS (**δAA 0.0225**, **(**0.050, *26***))**	
*Hypertensive (y/n)				**0.0076** (0.045, *224*)				NS (**δGG 0.0162**, **(**0.102, *49***))**
Systolic BP (recumbent mm Hg)	NS (**†Tt 0.0299,** 0.048, −0.355, 0.161, *98***)**				NS (**†AA 0.0412,** 0.070, −0.837, 0.401, *60***)**	NS (**†Bb 0.0290,** 0.054, −0.355, 0.160, *89***)**		NS (**†AA 0.0018,** 0.140, −0.655, 0.201, *67***) and** (**†GG 0.0304,** 0.096, 1.237, 0.554, *49***)**
Diastolic BP (recumbent mm Hg)				NS (**†CC 0.0457,** 0.098, 0.405, 0.196, *41***)**			NS (**†AA 0.0215,** 0.209, −0.402, 0.163, *25***)**	

^a^*P*-values are for standard least squares regression except for * where an effect likelihood ratio test has been conducted (in all cases *r*^2^ and the number of observations are given in brackets). † implies genotype influences the relationship between dietary vitamin D and phenotype; values show *P*, *r*^2^, slope estimate, SE and in italics the number of observations (*δ* implies the same vitamin D influence on the relationship, but for categorical data).

Again, the VDR-BsmI and VDR-TaqI seemed particularly relevant (especially in the context of the relationship between vitamin D and both insulin level and systolic blood pressure), making them important at both early and late lifecycle phases. However, other VDR genotypes were also clearly relevant, especially VDR-ApaI. Indeed, all variants showed a varying level of association with at least one of the clinical/biochemical phenotypes examined.

### Folate-related findings

Four folate-related gene variants were examined and are listed in Table 4. The table shows where any significant relationships existed between total sunspot activity (TSI) and genotype for each week of the first trimester. The table gives the *P*-value (including *r*^2^ and the number of observations) for the effect likelihood ratio following stepwise regression and subsequent ordinal logistic modelling. Similarly, Table 5 indicates where any significant relationship existed between photoperiod at conception and folate genotype occurrence.

**Table 4.** Significant relationships between total sunspot activity (TSI) and folate gene variants for each week of the first trimester of pregnancy.^a^

Post-conception week	19 bp del-DHFR	1494del6-TS	2R3R-TS	C1420T-SHMT
Week 1				
Week 2				
Week 3				
Week 4				
Week 5				
Week 6	*0.0025 (0.026, 229)			**0.0249** (0.012, 229)
Week 7			*0.0508 (0.009, 229)*	
Week 8				
Week 9				
Week 10	**0.0288** (0.026, 229)			
Week 11				
Week 12				

^a^The table gives the *P*-value (including *r*^2^ and the number of observations) for the effect likelihood ratio test following stepwise regression analysis and subsequent ordinal logistic fit. An asterisk indicates where significance is maintained following a Bonferroni correction for multiple comparisons.

**Table 5.** Significant relationships between photoperiod at conception and occurrence of genotype for four folate-related genes.^a^

	19 bp del-DHFR	1494del6-TS	2R3R-TS	C1420T-SHMT
Photoperiod at conception			**0.0360** (0.010, 221)	

^a^The table gives the *P*-value (including *r*^2^ and the number of observations) for the effect likelihood ratio test using ordinal logistic regression.

Table 4 illustrates that TSI had a significant effect on 19 bp del-DHFR and C1420T-SHMT occurrence, and that the greatest focus of this effect occurred during week 6 of pregnancy, almost mid-way through the first trimester. Table 5 indicates that only one of the four folate genes (2R3R-TS) was affected by photoperiod at conception. Interestingly, the 2R3R-TS gene was also borderline significant at week 7 for an effect of TSI on occurrence of genotype. Only the effect on19bp del-DHFR maintained significance following a Bonferroni correction for multiple testing (*P* = 0.0025).

The extent to which these folate gene variants influenced the same health phenotypes as those examined in relation to the VDR is given in Table 6, although the dietary effect of folate on this relationship is not given. Figure 2 shows the effect of TSI on 19 bp del-DHFR genotype occurrence for post-conceptional week 6. Table 6 indicates that C1420T-SHMT stood out as being the most relevant genotype in predicting important clinical phenotypes later in life, having significant associations with both fasting blood glucose and HbAIc.

**Table 6.** Later life biochemical and clinical phenotypes associated with folate gene variant occurrence.^a^

Clinical/Biochemical Phenotype	19 bp del-DHFR	1494del6-TS	2R3R-TS	C1420T-SHMT
Insulin (mIU/l)		*0.0544 (0.25, 229)*		
Fasting glucose (mmol/l)				**0.0037** (0.049, 225)
HbA1c (%)				**0.0297** (0.031, 228)
BMI				
Homocysteine (µmol/l)				
Cysteine (µmol/l)				
Glutathione (µmol/l)				
Red cell folate (nmol/l)				
*Cognition				
*Depression				
*Hypertensive (y/n)				
Systolic BP (recumbent mm Hg)				
Diastolic BP (recumbent mm Hg)				

^a^*P*-values are for standard least squares regression except for * where an effect likelihood ratio test has been conducted (in all cases *r*^2^ and the number of observations are given in brackets).

### Non-vitamin-related findings

Stepwise regression indicated that no direct relationship was evident between TSI at post-conception weeks 1–12 for fasting glucose, BMI, insulin, hypertension or systolic blood pressure in adulthood. However, when TSI at all post-conception weeks was regressed against HbA1c, week 4 gave a significant outcome (*P* = 0.0055, slope = −2.15 × 10^−^^5^). Similarly, TSI at post-conception weeks 5 and 10 was significant for cognition (*P* = 0.0044, slope = −7.83 × 10^−^^5^ and *P* = 0.0261, slope = 5.58 × 10^−^^5^, respectively), while at week 1 it was significant for depression (*P* = 0.0176 and 4.57 × 10^−^^5^). Solar irradiance at post-conception weeks 1 and 9 was also significant for recumbent diastolic blood pressure (*P* = 0.0016*, slope = 3.40 × 10^−^^4^ and *P* = 0.0210, slope = −2.40 × 10^−^^4^, respectively). Only one association* retained significance following a Bonferroni correction for multiple comparisons.

Blood metabolite levels are largely a product of diet and as such any relationship to irradiance/photoperiod at conception is unlikely.

Photoperiod at conception had no significant effect on fasting glucose, HbA1c, insulin, BMI, cognition or any of the blood pressure or metabolite measurements examined in adulthood. However, it did have a significant effect on depression in adulthood (*P* = 0.0240, slope = −0.00247), indicating that longer photoperiods at conception were associated with less depression in adults.

The final examination involved looking to see whether dietary intake of vitamin D was predictive of the clinically and biochemically relevant phenotypes listed in Table 3, independent of VDR genotype. In no cases was any association detected.

For convenience, and to provide the best overall summary of the data, the direction of effect for significant linear associations and response to genotype (highest to lowest) is given in Table 7.

**Table 7.** Summary table showing direction of association for linear relationships and response to genotype (highest to lowest) where significance has been detected.^a^

**Phenotype**	VDR-TaqI	VDR-Tru9I	VDR-FokI	VDR-NIaIII	VDR-ApaI	VDR-BsmI	VDR-Cdx2	VDR-EcoRV	1494del6-TS	C1420T-SHMT
Insulin (mIU/l)	+ve for vit D within tt				+ve for vit D within AA	+ve for vit D within BB			*6/6>6/0 >0/0*	
Fasting glucose (mmol/l)										TT > CT > CC
HbA1c (%)										TT > CT > CC
BMI		*+ve for vit D within Uu*	Ff ≥ FF > ff				+ve for vit D within GG			
Homocysteine (µmol/l)										
Cysteine (µmol/l)		uu > Uu > UU								
Glutathione (µmol/l)					+ve for vit D within aa			*AG > GG > AA*		
Red cell folate (nmol/l)										
*Cognition					−ve for vit D within aa					
*Depression					+ve for vit D within AA		+ve for vit D within AA			
*Percentage with hypertension				GG > GC > CC				−ve for vit D within GG		
Systolic BP (recumbent mm Hg)	−ve for vit D within Tt				−ve for vit D within AA	−ve for vit D within Bb		−ve for vit D within AA and +ve within GG		
Diastolic BP (recumbent mm Hg)				+ve for vit D within CC			−ve for vit D within AA			

^a^Italics designate where significance is approached. The use of the term ‘vit D’ refers to total dietary intake of the vitamin.

## DISCUSSION

Outcomes from this study can be broken down into five key relationships: (i) the periconceptional effect of duration and quanta of UV-R on occurrence of VDR and folate genotypes; (ii) the subsequent effect of VDR and folate genotypes on important clinical and biochemical phenotypes later in life; (iii) the VDR and folate gene-independent effect of periconceptional duration and quanta of UV-R on occurrence of important clinical and biochemical phenotypes later in life; (iv) the VDR-dependent and -independent effects of dietary intake of vitamin D on important clinical and biochemical phenotypes later in life and (v) the potential relevance of these findings in relation to the ‘folate/vitamin D sunshine hypothesis’ as a factor in the evolution of skin pigmentation. By looking at outcomes in this way, it may be possible to garner clues to some relevant early life events in the subsequent development of important adult phenotypes, including those which underscore disease processes. The clear association of vitamin D nutritional genetics and folate genes in any such phenomena justify significant discussion.

In 1962, James Neel introduced the idea of a ‘thrifty genotype’ and related it to diabetes—‘a thrifty genotype rendered detrimental by progress’ [73, 74]. The principle involved fat deposition at times of food surplus facilitating survival during famine. Hale and Barker were first to develop the concept of a ‘thrifty phenotype’ [75, 76]. They suggested that maternal undernutrition during pregnancy promotes metabolic adaptation that augments immediate survival, but since adaptations become fixed, they can modify tissue/organ structure, including hormone receptor density. The belief is that these changes remain advantageous in the long-term if nutritional environment is meagre [77], but become detrimental if nutritional environment is excessive, such as in today’s obesogenic environment [75, 76, 78–81]. Such events can increase the risk for type-2 diabetes in adults. Evidence now clearly indicates that many chronic adult disorders exhibit long latent periods that stem from the earliest phases of the lifecycle and which reflect developmental plasticity as an adaptive survival process. Epigenetic phenomena involved include DNA methylation and other histone modifications that regulate gene expression via chromatin remodelling. In this way, early life events that influence the genome and epigenome combine to shape adult phenotype.

### Periconceptional effect of duration and quanta of UV-R on occurrence of VDR genotypes, and relationship to important clinical and biochemical phenotypes later in life

The present study indicates that solar irradiance around conception influenced both VDR-BsmI, VDR-TaqI and VDR-EcoRV genotype occurrence through post-conceptional weeks 6–8 (a critical period when ossification begins) and that the first two of these variants, along with VDR-ApaI had a profound effect on insulin levels in adults when examined according to dietary vitamin D intake (Tables 1 and 3). There was no direct effect of periconceptional solar irradiance or photoperiod on adult insulin levels, nor was dietary vitamin D intake (independent of VDR genotype) associated with insulin levels. This is an interesting nutrigenetic relationship bound to a broader sphere of influence that includes the environmental influence of UV-R exposure during embryogenesis.

These findings are interesting because VDR-BsmI is related to osteocalcin level with the homozygote recessive (bb) genotype predicting a higher bone mineral density (BMD). Conversely, the wildtype (BB) genotype is linked to lower BMD in postmenopausal women [8, 9]. These findings have been repeated in numerous other publications since these initial reports [1], occasionally with conflicting results, which may stem from a variable diet–gene interaction [1]. The present data, as demonstrated in Fig. 2, shows that carriage of the mutant VDR-BsmI b allele increased as solar irradiance increased during post-conceptional week 7, a key time when bone mineralization is first initiated during development. Similarly, the VDR-TaqI T allele has been linked with increased risk for osteoarthritis of the knee [82], with concomitant work showing the *B*smI–ApaI–TaqI haplotype bAT is associated with reduced risk for osteoarthritis [83] and additionally, that the Bat and BAt haplotypes are significantly associated with osteoporosis [84, 85]. It is thus doubly interesting that in the present study, a similar gene–environment relationship existed between solar irradiance and both VDR-BsmI and VDR-TaqI through post-conceptional weeks 7–8—a crucial period in the developmental biology of bone tissue.

Intriguingly, there may be evidence that insulin, acting as an anabolic agent in bone, can preserve and increase bone density and bone strength, presumably through direct and/or indirect effects on bone formation [86]. With this in mind, the present data fits a model in which the VDR-BsmI b allele occurrence is associated in a positive way with UV-R exposure when embryonic ossification commences, and in later life, the B allele (BB genotype) is highly dependent on dietary vitamin D for maintaining insulin levels—the bb genotype is not (Tables 3 and 7). An important question is therefore whether VDR-BsmI genotype influences vitamin D reliant metabolism in a UV-R-dependent fashion, modifying an individual’s dependency for a dietary source of the vitamin through its effect on endogenous insulin production, insulin sensitivity, and insulin signalling, particularly with respect to bone. Unfortunately, we have no information on periconceptional insulin levels to develop this idea any further, but this perspective may have relevance to the developmental origins of adult disease, and is worth further consideration.

### Periconceptional effect of duration and quanta of UV-R on occurrence of folate genotypes and relationship to important clinical and biochemical phenotypes later in life

Folate plays an even more critical role in reproductive processes than vitamin D given its central role in dTMP synthesis and maintenance of the methylome. This latter process makes it an important mediator of early lifecycle adaptations that confer developmental plasticity. However, expression products of the folate gene variants examined here are primarily concerned with dTMP synthesis, with *de novo* methyl group synthesis being a metabolic downstream phenomenon. Since SHMT, TS and DHFR represent a specific gene cluster that encodes proteins operating in close synergy in respect of DNA synthesis, the finding that three of the four variant genes (19 bp del-DHFR, C1420T-SHMT and to a lesser extent, 2R3R-TS) were associated with TSI at around post-conceptional week 6 in particular, is extremely interesting. Given potential inaccuracy over the precise point of conception, the time frame for this effect may extend into a period that covers the occurrence of early malformations such as NTD. The timing of the environmental effect (UV-R exposure) on the folate and VDR genes is therefore consistent with what is already known about (i) the critical timing of periconceptional folate to prevent NTD (neural tube closes around 1 month post-conception) and (ii) the role of vitamin D in bone mineralization which begins at around week 7 of pregnancy. This could be coincidence, but equally may reflect the importance of these genes, and sensitivity of their variant forms, to environmental stimuli at key stages in embryo development.

In terms of folate genetics, it is interesting to note that of the adult phenotypes examined, only C1420T-SHMT had a significant effect, being associated with both fasting glucose and HbA1c.

### VDR and folate gene-independent effect of periconceptional duration and quanta of UV-R on occurrence of important clinical and biochemical phenotypes later in life

VDR and folate gene-independent effects of periconceptional solar irradiance/photoperiod on occurrence of important clinical and biochemical phenotypes later in life were detected. TSI at post-conceptional week 4 was significantly associated with HbA1c, TSI at weeks 5 and 10 was significantly associated with cognition, and TSI during weeks 1 and 9 was associated with indices of hypertension and depression (week 1 only in the latter case). Photoperiod at conception was most notable in its association with depression in adulthood.

### VDR-dependent and -independent effects of dietary intake of vitamin D on important clinical and biochemical phenotypes later in life

There was no gene-/photoperiod-/TSI-independent effect of dietary vitamin D on any clinically or biochemically relevant phenotype. However, dietary vitamin D seemed to show a significant interactive effect with VDR gene variants in respect of many important late life phenotypes. As mentioned above, Table 3 shows that VDR-BsmI, VDR-TaqI and VDR-ApaI genotypes had a profound effect on insulin levels in adults when examined according to dietary vitamin D intake. These same VDR variants also interacted with dietary vitamin D to modify systolic blood pressure as did VDR-EcoRV. In contrast, dietary vitamin D modified diastolic blood pressure according to VDR-Cdx2 and VDR-NIaIII variants. However, when considered as a categorical index (hypertensive or non-hypertensive), only VDR-EcoRV seemed to interact with vitamin D. Previously reported findings related to vitamin D and blood pressure are inconsistent; an association has been shown to exist between VDR-BsmI genotype and blood pressure in healthy men. In this case, higher blood pressure was linked to the polymorphic b allele [87]. However, the opposite outcome has also been published [88]. Others have reported susceptibility to calcific aortic valve stenosis in individuals with the B allele [89], but no relationship between the BsmI polymorphism and coronary artery disease [90], although an increased susceptibility to myocardial infarction in association with the B allele has been reported [91]. These findings were consistent with a report of the BB genotype being associated with a higher intimal-medial carotid artery thickness [92]. A recent review of the literature concludes a clear link does exist between low vitamin D levels and cardiovascular health, with cross-sectional data showing associations between low vitamin D and stroke, myocardial infarction, diabetes mellitus, hypertension and heart failure, with longitudinal data indicating a relationship with incident hypertension and recent cardiovascular events [93].

Table 3 also shows that VDR variants Cdx2 (GG) and possibly Tru91 (Uu) interacted with dietary vitamin D to modify BMI, while VDR-Fok1 was significantly associated with BMI independent of dietary vitamin D. This, and the relationship with insulin described above for the Taq1, Apa1 and Bsm1 variants is consistent with published data suggesting a link between vitamin D status altered insulin synthesis and secretion [3] and an association with obesity [4]. However, as others have alluded to, the nutrient–gene–phenotype interactions are clearly very complex, with equivocal results being published [1]. Another major finding here is that VDR variants ApaI (AA) and Cdx2 (AA) interacted with dietary vitamin D to modify likelihood of depression. Again, it is accepted that low vitamin D concentrations are associated with depression, and the evidence for this is given in a recent systematic review and meta-analysis [94].

### Potential relevance of findings in relation to the ‘folate/vitamin D sunshine hypothesis’ as a factor in the evolution of skin pigmentation

It is interesting to consider the present findings in a more holistic context; one that not only examines early, prenatal environmental influences on nutrient-related genes that can influence human disease later in life, but that also explores how such early events might have shaped the phenome in terms of the evolution of skin pigmentation. Jablonski and Chaplin [44] suggest melanization is the result of two clines produced by natural selection to adjust levels of constitutive pigmentation to accommodate the prevailing level of UV-R. They postulate that one cline originates from high UV-R near the equator and led to the evolution of a dark and photoprotective, eumelanin-rich skin pigment. The second cline involved depigmentation to allow for vitamin D synthesis at latitudes where UV-R is low. As humans spread out of the tropics, selection pressures based on the need to protect UV labile folate, and allow vitamin D synthesis, both required for reproduction, helped maintain the appropriate level of pigmentation, including facultative pigmentation (tanning) at latitudes where UV-R varies by season. The effects of UV-A and -B on folate and vitamin D, and the strength of this effect on natural selection have been detailed by Jablonski and Chaplin [44]. The present study is the first to examine the association between periconceptional UV-R exposure and occurrence of folate and vitamin D gene variants that might influence embryonic survival under adverse nutritional conditions. These findings may therefore further support the ‘folate–vitamin D-sunlight hypothesis’ based on the UV-R selection of key gene variants.

SHMT, DHFR and to a lesser extent, one of the TS variants are significantly associated with TSI during the first few weeks of embryogenesis. The expression products have a critical role in folate-dependent thymidylate biosynthesis, with SHMT of particular interest. SHMT has a crucial role in the repair of UV-induced DNA damage [30]. SHMT expression levels and post-translational modification of TS (SUMOylation) increase as does the nuclear compartmentation of SHMT and TS following UV-R exposure. Although this SHMT-related UV-R response occurs in humans, it does not occur in mice [95], indicating a clear species specificity and hence the possibility that it may have evolved as an adaptive response to protect skin from UV damage. It may therefore be a relevant phenomenon in considering the ‘folate–vitamin D-sunlight hypothesis’. TS is also responsible for dTMP synthesis, and it is well established that thymine bases and hence dTMP within DNA are highly sensitive to UV-R-induced damage. The nature of the damage is varied, but typically involves the formation of cyclobutane-pyrimidine dimers and various photoproducts [96] which hinder transcription. These photo-lesions are repaired via nucleotide excision repair (NER), although where an unfavourable ‘gene–nutrient–UV’ permutation occurs, the reduced dTMP pool and excess NER activity could lead to DNA fragility via excess uracil misincorporation. UV-R stimulated SHMT synthesis counters this, but highlights how common gene variants within this complex enzyme cluster might modulate the cellular response to UV-R. This effect of genetic variation would be further modified by the cellular folate status, because 5,10CH_2_-H_4_PteGlu supplies the specific one-carbon unit needed for dTMP synthesis. Maintenance of the reduced folate pool for continued dTMP synthesis is entirely dependent on the action of DHFR. Folate is itself highly sensitive to UV-R, and given the very early post-conceptional effects on DHFR, SHMT and TS seen here, and critical role of folate at this time in pregnancy, it is hard to dismiss the possibility that UV-R exposure could influence selection of specific DHFR, SHMT and TS genotypes.

Figure 2 illustrates the folate variant genotype prevalence according to TSI exposure. It shows that at post-conceptional week 6, the 19 bp del-DHFR 1/1 genotype was associated with the highest TSI (*P* = 0.0025), while for C1420T-SHMT, carriage of the mutant allele was associated with the greatest TSI (*P* = 0.0249). Although occurrence of 2R3R-TS genotype is only approaching significance at week 7, it is still worthy of attention. Protein expression from the 2R/2R-TS variant is 3- to 4-fold less efficient compared with the 3R/3R variant [60, 97]. It is of further interest that the frequency of the 2R/2R genotype varies by ethnicity; highest frequencies have been recorded in white populations (19–23%) [98–100], a lower prevalence has been recorded in Africans (14–19%) [101] and a very low prevalence of only 2–10% has been observed in Asian populations [102–105]. Whether this has any bearing on UV-R exposure and degree of skin pigmentation is unclear, but it may add weight to the putative link between this gene and the ‘folate–vitamin D-sunlight hypothesis’. The C1420T-SHMT maternal T allele confers a doubling in risk of preterm birth, although this was only shown in white American women. The same study did not observe this risk in African-American women, except in women who had a combination of low folate intake and the T allele [106]. Also, a similar pattern of ethnic prevalence to the 2R3R-TS 2/2 genotype exists; the frequency of the SHMT TT genotype is 7–13% in white populations [62, 106–109], 6.7% in African-Americans [106] and 0.2–1.7% in Asians [103]. Again, the question arises as to whether pigmentation might influence genotype occurrence.

An intriguing aspect of Fig. 2 is the similarity in the pattern exhibited for the 2R3R-TS genotype according to TSI exposure at post-conceptional week 7 (*P* = 0.05), and that for 2R3R-TS genotype by photoperiod at conception (*P* = 0.036). Whether this has any bearing on DNA and/or folate damage is unclear, although it may be relevant to the DNA repair mechanisms alluded to above (i.e. increased SHMT expression via a UV-responsive internal ribosome entry site that enhances DNA repair [30]).

Most research on UV exposure and DNA damage is linked to photo ageing and carcinogenesis. It is in this context that cellular protective responses including cell cycle arrest, DNA repair and apoptosis are most often considered; however, we propose that very early prenatal exposure to UV-R may play a role in defining critical genotypes (DHFR, SHMT and TS) that influence the competency of DNA synthesis and maintenance. These same genes may interact with UV exposure according to skin melanin levels, and influence evolutionary aspects of the pigmentation phenotype.

The critical nature of folate for DNA metabolism around the time of conception is clear, but there is perhaps less evidence for the role of vitamin D at this time point, with fewer studies having been conducted. However, higher serum and follicular fluid 25(OH)D_3_ levels have been shown to predict success in *in vitro* fertilization techniques [110]. Furthermore, during pregnancy, maternal vitamin D requirements increase 4- to 5-fold to facilitate the extra calcium required for skeletal growth, and hence women at high latitudes during winter and/or who have effective barriers to cutaneous UV-R exposure (dark pigmentation/sunscreen/veiled) are at increased risk of vitamin D insufficiency [14]. Additionally, there may be longer term effects on reproductive success—vitamin D deficiency during the first few years of life can result in a malformed pelvis, making it difficult for childbirth [111]. Despite this, few VDR-related effects have been examined early in the lifecycle, during embryogenesis, with most research focusing on how VDR modulates expression of proteins that are involved in signalling, intestinal calcium and phosphate absorption, and calcium homeostasis with the emphasis on the chronic diseases of ageing, particularly osteoporosis, cancer, diabetes, arteriosclerosis, CVD and infection. It is therefore unclear how relevant the TSI around conception is in terms of selecting VDR genes that might influence/support an evolutionary paradigm for skin pigmentation. However, because both VDR-BsmI and VDR-TaqI genotype occurrence through post-conceptional weeks 7–8, and VDR-EcoRV in week 6 were related to TSI, and because this is a critical period when ossification begins, it may be that evolutionary pressures can operate via the VDR to influence embryo survival. Given the global influence of VDR on homeostasis, other, yet to be discovered, critical early life events may also be modulated by vitamin D responsive elements.

The occurrence and nature of the folate and VDR genes that are affected add weight to the role of vitamins in the evolution of skin pigmentation, but in the context of vitamin D in particular, the influence of TSI on VDR genotype occurrence and subsequent effect of VDR (including the interaction with dietary vitamin D) on later lifecycle diseases seems especially relevant. It is also important to recognize the relevance of other genes in the evolution of skin pigmentation/depigmentation. As compelling as the putative role of vitamins and vitamin-related genes might be in skin pigmentation, it is important to temper this model with other recent findings: SLC24A5 seems especially important in the evolution of depigmentation in Europeans but not Asians [112]. Most Africans and East Asians carry one of two variant alleles for this gene, whereas 98% of Europeans carry the other variant. The report suggests that depigmentation in Europe occurred as recently as the past 6000–12 000 years. The possible selection pressure for this could still be vitamin D related though, with a move away from fishing and hunting that provided preformed dietary vitamin D, to a farming culture with fewer sources of preformed dietary vitamin D [113]. Additionally, other genes contribute to a multifactorial involvement in maintaining pigmentation; MC1R, MATP (SLC45A2), OCA2, TYRP1, DCT, KITLG, PPARD, DRD and EGFR are all likely to be relevant [27, 38, 41–43]. Some loci such as MC1R are strongly ‘committed’ to pigmentation, but many other loci that contribute to pigmentation variation also contribute to many other functions and systems in the body—natural section determines pigmentation phenotype, and in achieving this, simply uses the genes that are available.

**Figure 3.**
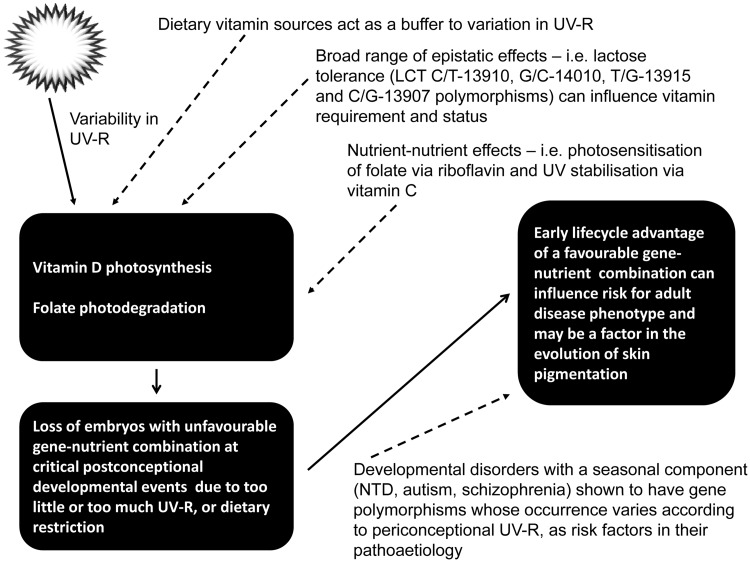
Simple schematic demonstrating how environmental and nutritional agents that interact to modify gene–phenotype relationships across the lifecycle might operate

The involvement of vitamin D in human ecology and disease aetiology is particularly complex because of the multiple pathways involved. We have demonstrated that VDR polymorphisms affected by TSI early in life also determine the relevance of dietary sources of vitamin D in defining insulin levels later in life. This novel finding fits well with a very recent study that shows UV-R modulates the expression of an epigenome involving energy conservation [114]. In their research, the authors indicated that decreasing UV-R at conception and early gestation stimulates energy conservation in persons considered ‘diabetic’ in today’s calorie-rich, obesogenic nutritional environment. However, despite the apparent parallel track of such research, one cannot ignore more remote components that play an important role in vitamin D, evolution and health. For example, lactose intolerance in dark-skinned African-Americans and Hispanics leads to reduced milk consumption compared with Europeans who developed the ability to metabolize lactose over time due to the development of dairy farming. Indeed, 70% of African-Americans, 53% of Mexican-Americans and 90% of Asians are lactose intolerant, compared with only 15% of northern Europeans [115]. Although milk is only a moderate source of both folate and vitamin D [116], the corollary of this is that a great many people may be at increased risk of deficiency in either or both vitamin because they are lactose intolerant; this is likely to be the case even where milk is a good source of vitamin D because the milk has been fortified with the vitamin. While the findings reported here focus on two tight gene clusters and a small number of adult phenotypes, it is likely that a very wide nutrigenetic circuit/net is relevant to the role of these vitamins, and particularly vitamin D in maintaining several homeostatic loci (see Fig. 3).

### Study limitations

This study attempts to examine environmental and nutritional agents that modify gene–phenotype relationships across the lifecycle, with the objective of providing new insight into human ecology. There are some drawbacks to our approach; observational study design, numbers are relatively small, the subjects’ maternal nutritional information relating to folate and vitamin D is absent, as is the genetic profile for both mothers and fathers of the study subjects, and gestation times can vary. Additionally, no accurate personal measure of exposure to UV-R, the type of clothing worn, physical activity levels, frequencies of outdoor activities and whether or not subjects’ parents had a healthy diet, exists. The data presented here are therefore entirely correlative, and despite having evidence for statistically significant relationships between the response variable and predictor variable(s) (*P* < 0.05), *r*^2^ values are sometimes low. Typically, around 5–10% of the variation in the response variable can be explained by the regression model, depending on the nature of the relationship examined, however, this does increase to around 40% in some analyses. We have applied a Bonferroni correction for multiple comparisons, and significance is still achieved for key folate and VDR genes, despite arguments against use of this test in exploratory data where potential relationships could be missed.

Despite these perceived limitations, signatures within the data emerge that indicate a novel ‘cradle to grave’ perspective linking early lifecycle environment with nutritional genetics that have a significant impact on risk for adult disease and disease risk factors. Unfortunately, one of the major effects in adults is likely to relate to BMD, and we have no measure of this in our subjects. This is perhaps the major study limitation. Another possible avenue for future research might involve measuring blood 25(OH)D_3_ and genetic loci (SNPs) in vitamin D binding protein that are known to affect 25(OH)D_3_ concentrations. Furthermore, it would be extremely useful to examine any association between skin type and genetic variants in relation to duration or quanta of UV-R. Additionally, some gene variants may influence disease susceptibility, and as a result the allele prevalence might shift between early and late lifecycle. Again, we have no information on this for the population being investigated here.

## CONCLUSIONS

The quanta and duration of UV-R exposure during the periconceptional period were associated with the occurrence of important vitamin D and folate-related gene variants. TSI was significantly associated with occurrence of VDR-TaqI, BsmI and EcoRV between post-conceptional weeks 6 and 8, a period when skeletal ossification begins, a physiologic process closely linked to UV-R-derived vitamin D synthesis and dietary intake. Similarly, TSI was significantly associated with occurrence of 19 bp del-DHFR, 2R3R-TS and C1420T-SHMT between weeks 6 and 7. This is a period recognized as being sensitive to a low folate and risk of congenital malformation. As folate is UV labile, low levels may be particularly sensitive to UV-R exposure during the periconceptional period. Photoperiod at conception also seems to predict VDR-Tru9I and 2R3R-TS genotype.

These genes that appear to be sensitive to environmental stimuli early in life are also clearly linked to important clinical and biochemical phenotypes later in life. In particular, dietary vitamin D strongly predicted blood insulin level according to VDR-TaqI (tt) and BsmI (BB) genotype. A range of other adult phenotypes were predicted by VDR in a similar way (see Tables 3 and 7). Folate gene variants were not examined according to dietary intake, but C1420T-SHMT genotype showed a significant relationship with both fasting blood glucose level and percentage HbA1c. In both cases, the homozygote recessive (TT) genotype had the higher index, followed by the heterozygote genotype (CT) and then the wildtype (CC).

One of the most interesting considerations relates to whether the variant VDR and folate gene correlation to periconceptional UV-R might reflect a mechanism that has relevance to the evolution of skin pigmentation. The data could fit with Jablonski and Chaplins view [44] that melanization is the result of two clines produced by natural selection in order to adjust levels of constitutive pigmentation to accommodate the prevailing level of UV-R. The 2R3R-TS 3/3 genotype was associated with the highest TSI and longest photoperiod. Precisely how UV-R, folate and 2R3R-TS interact is unclear, although in the case of C1420T-SHMT, a higher TSI was associated with carriage of the T allele, which is linked to adverse pregnancy outcomes in the face of a low blood folate [106]. Perhaps UV-R-related DNA (and/or folate) damage renders the early embryo sensitive to specific folate-related genotypes whose expression products are critical to DNA synthesis and repair. Under these circumstances, this paradigm for the evolution of skin pigmentation seems reasonable, although a better understanding of the functional consequences of these folate polymorphisms is needed to be certain.

The observed effect of periconceptional TSI on VDR-TaqI, BsmI and EcoRV could also support this model for the evolution of skin pigmentation. Clearly, the multiple effects of the VDR mean there are many potential ways in which UV-R might interact with this polymorphic gene to influence embryo viability and fit the hypothesis. However, in the context of the present study, this may best relate to calcium metabolism and early skeletal development. The VDR-BsmI bb genotype is known to be associated with increased BMD [8, 9], and we have shown that the highest TSI was associated with this recessive bb genotype. Thus, vitamin D synthesis would be favoured under TSI conditions that also favour occurrence of the genotype linked to optimal bone mineralization, and at a time when ossification of the skeleton first occurs. So once again it seems that under these circumstances, this paradigm for the evolution of skin pigmentation appears reasonable, although to be certain of this, the functional consequences of the VDR polymorphisms need to be elucidated as is the case with the folate variants discussed earlier.

### Concluding summary points

1. TSI was significantly associated with occurrence of VDR-TaqI, BsmI and EcoRV between post-conceptional weeks 6 and 8, a period when skeletal ossification begins, a physiologic process closely linked to UV-R-derived vitamin D synthesis and dietary intake.
2. TSI was significantly associated with occurrence of 19 bp del-DHFR, 2R3R-TS and C1420T-SHMT between weeks 6 and 7, a period recognized as sensitive to low folate and hence risk of congenital malformation.
3. Photoperiod at conception predicts VDR-Tru9I and 2R3R-TS genotype.
4. Dietary vitamin D strongly predicted blood insulin level according to VDR-TaqI (tt) and BsmI (BB) genotypes. (Tables 3 and 7 list several other adult phenotypes predicted by VDR genotype).
5. C1420T-SHMT genotype showed a significant relationship with both fasting blood glucose level and HbA1c.
6. The correlation of variant VDR and folate genes with periconceptional UV-R exposure might reflect a mechanism that has relevance to the evolution of skin pigmentation.

## IMPLICATIONS

Findings identify environmental and nutritional agents that interact to modify gene–phenotype relationships across the lifecycle, offering new insight into human ecology. Results support and extend previous findings relating to UV and folate gene selection [27, 28]. It also lends support to the role of these two vitamins in the evolution of skin pigmentation, providing a context to how UV-R might interact with polymorphic vitamin D/folate genes to influence embryo viability.

**Conflict of interest**: None declared.
